# Supplementary figures and images for: Appropriate-for-gestational-age infants who exhibit reduced antenatal growth velocity display postnatal catch-up growth
Source: PLoS One. 2020 Sep 8;15(9):e0238700. doi: 10.1371/journal.pone.0238700 (PMC7478563; doi:10.1371/journal.pone.0238700)

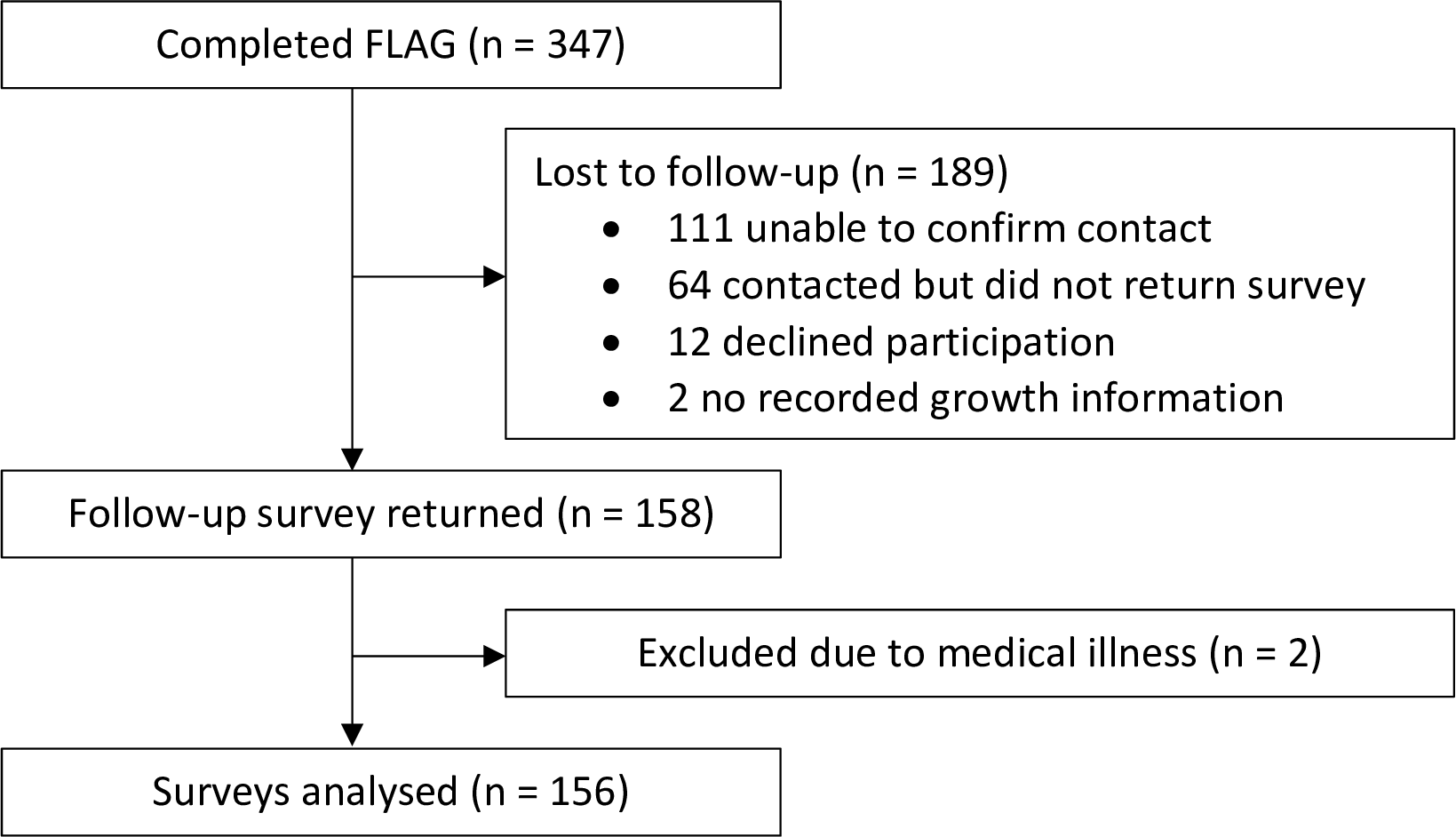

Supplement: S1 Fig — (TIF) [file pone.0238700.s001.tif]

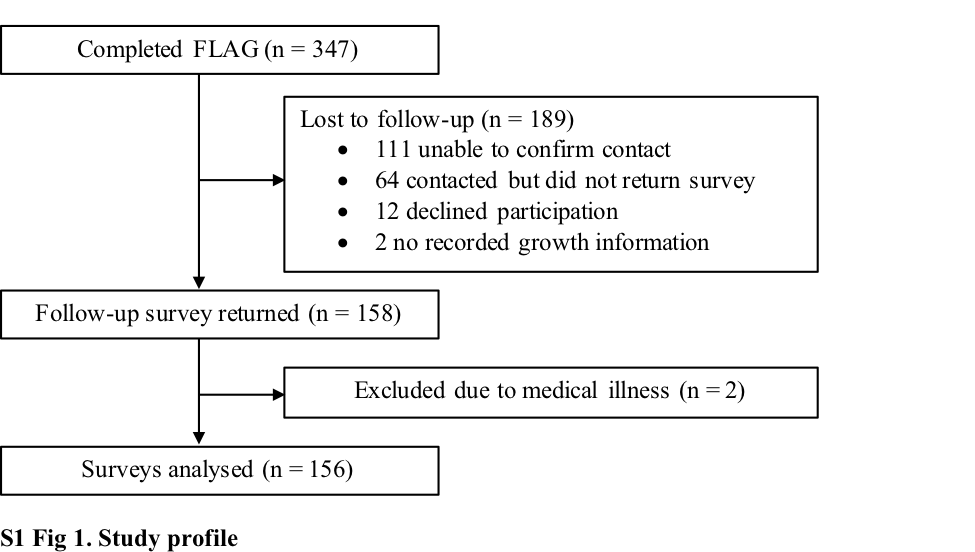

Supplement: S1 File — (ZIP) [file pone.0238700.s005.zip › Supporting information Final/S1_Fig.tiff]
