# Supplementary material for: Appropriate-for-gestational-age infants who exhibit reduced antenatal growth velocity display postnatal catch-up growth
Source: PLoS One. 2020 Sep 8;15(9):e0238700. doi: 10.1371/journal.pone.0238700 (PMC7478563; doi:10.1371/journal.pone.0238700)
Supplement: S1 Table — (DOCX) [file pone.0238700.s002.docx]

**S1 Table 1. Demographic and delivery characteristics of responding participants** **compared to eligible women who did not respond.**

|  | Responders  (n = 158) | Non-Responders  (n = 189) | *P* |
| --- | --- | --- | --- |
| Age (years) | 31.5 (3.9) | 30.3 (4.3) | 0.004* |
| Booking BMI (kg/m^2^) | 23.6 [21.1-26.6] | 23.85 [21.6-27.6] | 0.090 |
| Smoking status |  |  | 0.73 |
| Current smoker  Ex-smoker  Never  No info | 2 (1%)  40 (25%)  115 (73%)  1 (1%) | 4 (2%)  53 (28%)  132 (70%)  0 (0%) |  |
| Gestational hypertension  or pre-eclampsia | 20 (12.7) | 29 (15.3) | 0.54 |
| GDM | 12 (7.6) | 31 (16.4) | 0.01* |
| Mean EFW centile change in 8 weeks (centile/8 weeks) | -4.8 (21.3) | -6.1 (21.6) | 0.57 |
| EFW change >20 centiles | 40 (25%) | 49 (26%) | 0.90 |
| Onset of labour |  |  | 0.02* |
| Spontaneous labour  Induction of labour  No labour | 80 (51%)  70 (44%)  8 (5%) | 70 (37%)  100 (53%)  19 (10%) |  |
| Mode of delivery |  |  | 0.13 |
| Normal vaginal delivery  Instrumental delivery  Emergency caesarean  Elective caesarean | 68 (43%)  52 (33%)  31 (20%)  7 (4%) | 63 (33%)  63 (33%)  46 (24%)  17 (9%) |  |
| Gestational age at delivery (weeks) | 39.57 [38.8-40.5] | 40.00 [38.9-40.8] | 0.15 |
| Birthweight (kg) | 3.41 (0.5) | 3.329 (0.5) | 0.12 |
| WHO birthweight centile | 59.9 [30.0-80.4] | 52.0 [27.5-79.7] | 0.21 |
| Customised birthweight centile | 41.5 [23.1-66.0] | 38.8 [16.3-71.5] | 0.30 |
| SGA (<10^th^ WHO centile) | 10 (6%) | 19 (10%) | 0.25 |
| SGA (<10th customised centile) | 10 (6%) | 29 (15%) | 0.01* |

Data presented as mean (standard deviation) or median [interquartile range] depending on distribution for continuous variables, and as number (%) for categorical variables

BMI body mass index; GDM gestational diabetes mellitus; EFW estimated fetal weight; WHO World Health Organisation; SGA small-for-gestational-age; * p<0.05
