# Supplementary material for: Appropriate-for-gestational-age infants who exhibit reduced antenatal growth velocity display postnatal catch-up growth
Source: PLoS One. 2020 Sep 8;15(9):e0238700. doi: 10.1371/journal.pone.0238700 (PMC7478563; doi:10.1371/journal.pone.0238700)
Supplement: S1 Appendix — (PDF) [file pone.0238700.s003.pdf]

S1 Appendix. Patient Questionnaire

Patient Questionnaire – FLAG study follow-up

Mercy Hospital for Women, Heidelberg

Principal Researcher: Professor Sue Walker

Associate Researchers: E Mclaughlin, Dr T MacDonald

Personal Information

Name (mother):

Mercy UR No. (mother):

Name (child):

Mercy UR No. (child):

Sex (child):

Has your child been <u>admitted</u> to hospital since birth?

Yes ☐ No ☐

If yes:

Name of hospital:

Child's UR number (if known):

Age at admission (months):

Length of admission (days):

Medical problem leading to admission:

Has your child required any <u>ongoing follow-up</u> with a health practitioner since birth?

Yes ☐ No ☐

If yes:

Type of medical practitioner:

If "other specialist" please specify type:

Medical problem leading to ongoing follow up:

Follow up institution/clinic name

Growth Information

How to fill in the table:

Look at the growth charts in your child's My Health, Learning and Development Record' or 'Green Book'. To the best of your ability, enter in the measurements into the table below. You may not have measurements for every age, so just enter as many as you have depending on how old you child is. Please note that all data collected for this project will be de-identified, and participation is voluntary. Alternatively, you can take a photo of or scan the relevant growth pages of your child's book.

**Age of Infant**	**Date of Assessment** (dd/mm/yy)	**Weight (kg)** e.g. 3.145	**Length (cm)** e.g. 65.3	**Head Circumference (cm)** e.g. 35.5
HOME VISIT				
2 WEEKS				
4 WEEKS				
8 WEEKS				
4 MONTHS				
6 MONTHS				
8 MONTHS				
12 MONTHS				
18 MONTHS				
2 YEARS				

How to return the consent, questionnaire and/or photos:

- **Post**: C/O FLAG Study, Mercy Perinatal, Level 3, Mercy Hospital for Women, 163 Studley Rd, Heidelberg VIC 3084, Australia
- **Email**: flagstudy@mercy.com.au
