# Supplementary material for: Appropriate-for-gestational-age infants who exhibit reduced antenatal growth velocity display postnatal catch-up growth
Source: PLoS One. 2020 Sep 8;15(9):e0238700. doi: 10.1371/journal.pone.0238700 (PMC7478563; doi:10.1371/journal.pone.0238700)
Supplement: Attachment — Submitted filename: Response to Reviewers.docx [file pone.0238700.s006.docx]

Dear Editors of PLOS One,

**Re:	PONE-D-19-31762**

**McLaughlin et al, ‘Appropriate-for-gestational-age infants who exhibit reduced antenatal growth velocity display postnatal catch up growth’**

We thank the reviewers for their feedback and suggestions, which have improved the manuscript. Major statistical revisions have been performed for this resubmission as requested. Here we are pleased to provide our point-by-point responses, and submit this revised manuscript for consideration.

For ease of review, we submit a manuscript with tracked changes, and also submit an identical clean copy. In this response letter, all page numbers and line numbers refer to those of the tracked changes copy.

We look forward to your further correspondence,

Dr Emma McLaughlin

Corresponding author.

**Reviewer #1**

1. ***Abstract:* While the Flag study used a more informed measure of EFW (used the slowing of growth) however this is not identified in the abstract and paper introduction and the abstract introduction only refers to weight <10th percentile.**

We have now added detail to the definition of “slowing growth” in the Background section of the abstract. Please see page 2, lines 22-24 which now read:

*“Infants who demonstrate slowing growth during pregnancy are those that, cross estimated fetal weight centiles at serial ultrasound examinations.”*

Detail regarding the definition of slowing growth is also given in the Methods section of the abstract. Page 2, lines 40-42 state:

*“Predicted mean age-adjusted z-scores were then compared between three groups; SGA, AGA with low antenatal growth (AGA-FGR, loss of >20 customised estimated fetal weight centiles)”*

2. **The abstract needs to mention the high loss to follow which was ~ 50%, and the high proportion of GDM in those lost to follow up compared to respondents.**

As requested, we have now included this information in the abstract, results section. We write, page 3, line 48:

*“Of 158 (46%) infant growth records received,…”* – hence reporting the follow up rate.

And on page 3, lines 49-50 we have added

*“Rates of gestational diabetes and SGA birthweight were higher in those lost to follow-up”.*

3. **The word “responses” was used in the abstract but it is not clear what it means.**

In response to this reviewer comment, we have now adjusted “responses” to “infant growth records” for greater clarity. (Abstract, Results subsection, page 3, line 48)

4. **The abstract needs to mention that the postnatal anthropometric measurements were obtained by parent report.**

The abstract has been altered to mention that the parents were asked to send the measurements, and also to clarify that the measurements themselves were taken by health professionals, not the parents themselves. It now reads, (Page 2, lines 33-35):

*“To assess postnatal growth, we asked parents to send their infant’s growth measurements, up to two years post-birth, which are routinely collected through the state-wide Maternal-Child Health service.”*

5. **The study makes the point that not all infants with a birthweight >10th percentile have avoided growth restriction. However, the counter problems exists as well, since the 10th percentile is an arbitrary statistical cut-off. Not all infants that are born SGA with a birthweight <10th percentile have utero placental insufficiency. The paper’s introduction should mention that some infants in the SGA category are small normal infants.**

To meet this request of the reviewer we have now added a line to the introduction. Please see page 4, lines 70-75

*“SGA… is the most commonly used proxy for FGR, as although not all SGA fetuses are growth restricted (some are constitutionally small), it does represent an at-risk cohort.*

*Being SGA is the greatest risk factor for stillbirth (3) but its legacy also extends into postnatal life among survivors.”*

6. **In contrast to the statement “strong evidence that the development of cardiometabolic disease in adulthood is related to early key growth periods”, some of these presumed effects may be seen due to the analysis methods used. It would be valuable to consider possible over adjustment as described in the following references:
   1. Kramer MS, Zhang X, Dahhou M, Yang S, Martin RM, Oken E, et al. Does fetal growth restriction cause later obesity? Pitfalls in analyzing causal mediators as confounders. Am J Epidemiol. 2017 Apr;185(7):585–90.
   2. Huxley R, Neil A, Collins R. Unravelling the fetal origins hypothesis: is there really an inverse association between birthweight and subsequent blood pressure? Lancet 2002 Aug;360(9334):659–65.
   3. Paneth N, Ahmed F, Stein AD. Early nutritional origins of hypertension: a hypothesis still lacking support. J Hypertens Suppl. 1996 Dec;14(5):S121–9.
   4. Tu Y-K, West R, Ellison GTH, Gilthorpe MS. Why evidence for the fetal origins of adult disease might be a statistical artifact: the “reversal paradox” for the relation between birth weight and blood pressure in later life. Am J Epidemiol. 2005;161:27–32.**

While this study is based on belief in the fetal origins of adult health and disease theory, the reviewer raises a valid point in that this theory is not universally accepted. As such we have:

- Removed the word “strong” from the statement quoted by the reviewer above (page 4, line 82)
- Added a section to the strengths and limitations section of our discussion which states (Page 18, lines 369-374):

*“Finally, this study interrogating fetal and postnatal growth rates is based upon the developmental origins of health and disease hypothesis. While this is a theory to which we ascribe, it is not universally accepted, with some research groups considering that the relationships between fetal growth and adult disease seen may be due to over-adjustment due to analysis method used (34-37)”*

7. **It should be noted that there is a poor association between estimated foetal weight and birthweight.**

In the limitations section of the Discussion we have now noted this by saying (Page 18, lines 374-377):

*“In addition, the limitations of the original FLAG study have already been discussed (15), but relevant here is that estimated fetal weight is only accurate to within 10% of the true weight 80% of the time (38).”*

8. **This study used percentiles to evaluate antenatal growth, but percentiles become meaningless at the extremes. SD scores, which were used in some analyses, are better than percentiles as they are meaningful over the full range of the values.**

In light of the comments from both Reviewer #1 and Reviewer #2, we have now repeated all of the analyses with the aid of a biostatistician Dr Richard Hiscock (MBiostat). All of the analyses have now been performed in using age-adjusted standard deviations z-scores rather than percentiles. You will see that the Methods and Results sections of the manuscript have both been largely edited.

9. **What precision was used to calculate the standard deviation scores, was it the WHO monthly data or daily data?**

We calculated infant age accurate to the number of days. When calculating z-scores we used age in months, accurate to precision of one decimal place – roughly equivalent to 3 days.

10. ***Analysis:* Were any variables adjusted for in the regression analysis? It is not necessary to adjust, in fact it is appropriate to report crude analyses, it just needs to be clear if adjustments were made in any of the analyses**

No adjustments were made. As such, we have clarified this in the methods, page 9, lines 206-207 where we now state:

*“The relationships between EFW growth velocity and postnatal weight, length and BMI were assessed in two ways, without adjustment for any other variables:”*

In addition, on page 10, lines 221-222 state:

*“No adjustment for multiple comparisons for either significance testing or confidence interval width was performed.”*

11. **Line 188: “Fishers exact test was used was used to ascertain the relative risk of catch up growth” does not make sense since it is a statistical test.**

This line (now Page 10, lines 214-222) has now been altered (it also reflects our updated statistical analyses performed) to read:

*“Secondly, we assessed rates and relative risks of catch-up growth between the three groups. Catch-up growth was defined as an increase in weight age-adjusted z-score of ≥0.67 between birth and 12 months of age (29). Analysis used Fisher’s exact test across the three antenatal growth categories. If the null hypothesis of no overall difference in proportions was rejected, between pair testing was performed.”*

**12. Results: While there were significant differences in birth size between the FGR & NG infants, several of the differences at birth were not clinically importantly different.**

In contrast to the reviewer, we take the view that a difference in mean birthweight of almost 20 centiles is clinically significant, in keeping with the point that those that slow in growth are growth restricted compared to their counterparts who maintain their EFW centile in utero. Nevertheless, Results, page 13, Lines 264-266 has been changed to specify ‘statistically significant’ when describing the differences seen, to maintain neutrality when presenting these results.

**13. Line 230 to 232 should be stated in the past tense since these results should not be generalized beyond the study, particularly since it is an observational study and one with a very large loss to follow up.**

This has been corrected to the past tense (now line 267-270, page 13).

**14. Line 33 and the abstract: “SGA infants … were excluded.” but SGA infants are included in the Results in line 244.**

Thank you for noticing this error. In the original FLAG study SGA infants were excluded from analysis, but they *were* included in the FLAG follow-up submitted here. As such, line 33 (Now line 35-36) has been corrected to only ‘infants with medical conditions’.

**15. Although widely used, it is important to mention that the “change in WAZ greater than or equal to +0.67 SD” has not been validated.**

This is a valid point. A sentence has now been added to our methods section, (Page 8, lines 182 to 184) to reflect this, where we state:

*“It is important to note however, that although widely used (30, 31), this definition of catch-up growth has not been formally validated.”*

**Reviewer #2:**

**McLaughlin et al present an interesting study on infants’ growth velocity according to their birthweight. I have the following comments, questions and suggestion:

1. Abstract: The abstract does not state clearly the aims of the study and the study design, that are instead specified at the end of the Introduction: line 99 “In this follow-up to the FLAG study we aimed to: i) determine whether AGA infants with slowing antenatal growth demonstrate catch-up-growth; ii) compare AGA catch-up growth to that of the SGA infants; and iii) determine in which postnatal time period catch up-growth occurs.”**

We have now adjusted the Background section of the Abstract to state more clearly the aims of the study as suggested by the reviewers. Page 2, lines 22-28 now read:

*“Infants who demonstrate slowing growth during pregnancy are those that cross estimated fetal weight centiles at serial ultrasound examinations. These infants that slow in growth but are born appropriate-for-gestational age (AGA; ≥10^th^ centile), exhibit antenatal, intrapartum and postnatal indicators of UPI. Here, we examine if and when these infants (labelled as AGA-FGR) also demonstrate catch-up growth like SGA infants, when compared with AGA infants with normal antenatal growth velocity (AGA-NG).”*

**2. The abstract does not specify the statistic methods utilized to reach the study goals.**

We have now detailed the statistic tests utilised in the abstract (Pages 2-3, lines 36-45) where we state:

*“From the measurements obtained we calculated age-adjusted z-scores for postnatal weight, length and body mass index (BMI; weight(kg)/height(m^2^)) at birth and 4, 8, 12, 18 and 24 months. We used linear spline regression modelling to predict mean weight, length, and BMI z-scores at intervals post birth. Predicted mean age-adjusted z-scores were then compared between three groups; SGA, AGA with low antenatal growth (AGA-FGR; loss of >20 customised estimated fetal weight centiles) and AGA-NG to determine if catch-up growth occurred. In addition we compared the rates of catch-up growth (defined as an increase in weight age-adjusted z-score of ≥0.67 over 1 year) between the groups with Fisher’s exact tests.”*

**3. Methods: Line 130: what is the rationale behind using a different cut off for growth velocity than the one used in the FLAG study ?**

We used a cut-off of >20 centiles EFW loss instead of >30 centiles loss to ensure enough cases for a reasonable statistical comparison, given that we expected loss to follow up. We have now added more detail to this point in the Methods section, page 7, lines 143-152 which reads:

***“****The current study focussed on comparing antenatal and postnatal growth trajectory, not predictive performance for adverse outcome for which the original study was powered. Given the different outcome being interrogated, and expected loss to follow up, we used a more sensitive cut-off for growth velocity than the original FLAG study to ensure a sufficient number of cases of low antenatal growth velocity. Fetuses who exhibited an antenatal decrease in EFW of >20 customised centiles over eight weeks were defined as experiencing low antenatal growth velocity and to potentially be growth restricted (labelled as AGA-FGR). The remainder of the AGA fetuses were defined as demonstrating normal antenatal growth (AGA-NG)."*

**4. Line 144: “the follow-up cohort was again divided into three groups: the SGA; the AGA with normal antenatal growth velocity (AGA-NG); and the AGA with low antenatal growth velocity (AGA-FGR)” This distinction into 3 study groups does not clearly appear from the abstract**

We have now adjusted the abstract background and methods to clarify the distinction into the three study groups.

The Background section of the abstract, page 2, lines 26-28 now states:

*“Here, we examine if and when these infants (labelled as AGA-FGR) also demonstrate catch-up growth like SGA infants, when compared with AGA infants with normal antenatal growth velocity (AGA-NG).*

The Methods section of the abstract, pages 2-3, lines 40-43 now state:

*“Predicted mean age-adjusted z-scores were then compared between three groups; SGA, AGA with low antenatal growth (AGA-FGR, loss of >20 customised estimated fetal weight centiles), and AGA-NG to determine if catch-up growth occurred.*

**5. Line 161: “To standardise growth velocity for the cohort, we divided the change in weight centile by the actual number of days between examinations and then multiplied by the exact number of days in that time epoch”. Could the authors clarify this part ?**

This sentence has been removed after inclusion of new statistical methods.

**6. Line 223 “We first analysed the relationship between antenatal growth velocity and postnatal growth velocity in AGA infants (Table 3). At birth there was a significant difference in weight (p=0.0002), length (p=0.01) and BMI (p=0.0002) centiles between the AGA-FGR and AGA-NG infants.” Why is this important? Isn’t such difference what discriminates between AGA-FGR and AGA-NG?**

AGA-FGR and AGA-NG are defined by their antenatal growth velocity and that they were born at birthweight ≥10^th^ centile, the groups are not defined by having different birthweight or other anthropometric measurements at birth. AGA-FGR infants, exhibited an antenatal decrease in EFW of greater than 20 customised centiles between 28- and 36-weeks’ gestation, while AGA-NG infants did not. Despite the difference in antenatal growth rate, both groups were born with weight greater than 10^th^ centile (AGA). Therefore, if we had not tracked antenatal growth velocity, they would be considered simply equal AGA infants. These initial differences in birth measurements are therefore important to note, as even by themselves they add weight to the evidence that AGA infants who have demonstrated slowing growth in utero are growth restricted/subject to uteroplacental insufficiency relative to their AGA counterparts who maintain their EFW centile throughout gestation. This was also seen in the initial FLAG study, but we found it prudent to report this again as evidence maintained even here among a different, smaller sub-cohort. In addition, that the infants of the AGA-FGR group are smaller at birth, and then no longer significantly smaller at 4 months of age is evidence of catch-up growth.

**Results:**

7. **If the authors state in line 144 state that they are interested in studying 3 groups, why do they only present data on 2 groups in table 2 ?**

SGA fetuses and infants are already known to be a cohort at increased risk. In contrast, AGA fetuses and infants who have demonstrated slowing antenatal growth are the cohort which in this study we primarily are investigating to see if they demonstrate catch-up growth, as further evidence of uteroplacental insufficiency compared to AGA fetuses who maintain their EFW centile across gestation. As such, we compared the maternal characteristics between AGA-FGR and AGA-NG groups.

This is now clarified in the Results section, page 11, lines 238-241 where we now state:

*“Given our primary question concerned whether AGA-FGR infants demonstrate catch-up growth compared to the AGA-NG, as a sign of UPI occurring among the AGA, we compared their maternal and pregnancy characteristics (Table 1).”*

8. **Table 3: again, why do the authors compare AGA-FGR and AGA-NG, instead of comparing the 3 groups AGA-FGR, AGA-NG, and SGA? As postnatal growth is assessed as a series of measurements on the same subject over time, the authors may consider statistical tools such as linear mixed models for longitudinal data instead of comparing differences between 2 time points at a time.**

In light of the reviewer’s comments, we have now reanalysed the data with different statistical methodology, see below.

9. **Table 4 considers 3 different study groups, why does table 3 consider only 2? Table 5: as the authors consider 3 groups, they should use a statistical test that investigates differences in multiple groups and then specify further sub-group differences using a post hoc test.**

After our reanalysis, we now present 2 tables of catch-up growth related data. Both include all three antenatal growth groups.

Table 3 (previously Table 5) still presents the results of between pair testing, but the results of testing the three groups together are presented in the text when we state (Results, page 16, lines 316-321):

*“When catch-up growth was assessed as a dichotomous outcome (change in WAZ ≥+0.67) between birth and 12 months of age between all three groups, a significant difference was found (p=0.004). When between pair testing was then performed, the SGA and AGA-FGR cohorts were both found to be at increased risk of catch-up growth when compared to the AGA-NG group (Table 3).*

10. **Personally, I would consider the 3 study groups based on birthweight (AGA-NG, AGA-FGR and SGA) and compare their postnatal growth with a linear mixed model. The model provides a coefficient that summarizes the change in weight percentile between different time points. The model would allow to compare the growth speed of AGA-NG, AGA-FGR and SGA infants and it would also enable to study subgroup differences. Furthermore, the model would also allow to control for confounding**

In response to Reviewer #2’s points 8-10 we have enlisted the expertise of biostatistician Dr Richard Hiscock (MBiostat) and we have reanalysed the data. You will note that the methods and the results sections of the manuscript have been rewritten to reflect these reanalyses.

Specifically, the Statistical Analysis section of the Methods now outlines the new analyses performed, reading (pages 9-10, lines 195-222):

### *“Antenatal growth velocity and catch-up growth*

*Growth curve modelling was performed using linear spline regression, to fit curves plotting the change in predicted mean weight, length and BMI z-scores over time, for each antenatal growth category. User determined knots were placed at five time points (4, 6, 12, 18 and 24 months). We also fitted growth curves using both: restricted cubic spline model with knots placed at the quartiles of the time distribution and an interaction between time and antenatal growth category; and locally weighted kernel regression. All statistical methods produced extremely similar results, reinforcing the validity of our linear spline regression approach (Data not shown, available upon request).*

*The relationships between EFW growth velocity and postnatal weight, length and BMI were assessed in two ways, without adjustment for any other variables. First predicted mean difference and associated 95% confidence limits for weight, height and BMI z-scores were calculated at birth, 2, 4, 6, 12, 18 and 24 months for the three comparisons between antenatal growth categories (SGA & AGA-FGR, SGA & AGA-NG, and AGA-FGR & AGA-NG). We defined catch-up growth as occurring if the SGA or AGA-FGR group infants were significantly smaller than the AGA-NG group infants in weight, length and/or BMI at one time point, followed by no significant difference in infant size months later. Secondly, we assessed rates and relative risks of catch-up growth between the three groups. Catch-up growth was defined as an increase in weight age-adjusted z-score of ≥0.67 between birth and 12 months of age (29). Analysis used Fisher’s exact test across the three antenatal growth categories. If the null hypothesis of no overall difference in proportions was rejected, between pair testing was performed. Statistical analyses were performed using GraphPad Prism software version 7.0d for Mac OS X (32) and the nonparametric series regression suite within Stata v16 (33). Significance level was two-sided and set at 0.05. No adjustment for multiple comparisons for either significance testing or confidence interval width was performed.”*

And the results presented reflect this analysis. We now include two new figures, Figures 1 and 2, which show the raw infant measurement data as well as the predicted means (and 95% confidence intervals) for infant weight, length and BMI for each of the three antenatal growth groups together.

11. **Discussion: Well written but reflects the weaknesses of the methodological design.**

We thank the reviewer most sincerely for these suggestions. In this revision we have tried to address the major weaknesses in design. In response to your suggestion we have conducted considerable further statistical analyses to compare all three groups. The additional statistical analyses performed are all outlined in the methods and results sections of the manuscript which are now considerably different to that of the manuscript originally submitted.
