# Supplementary material for: Appropriate-for-gestational-age infants who exhibit reduced antenatal growth velocity display postnatal catch-up growth
Source: PLoS One. 2020 Sep 8;15(9):e0238700. doi: 10.1371/journal.pone.0238700 (PMC7478563; doi:10.1371/journal.pone.0238700)
Supplement: Attachment — Submitted filename: Second response to Reviewers.docx [file pone.0238700.s007.docx]

Dear Editors of PLOS One,

**Re:	PONE-D-19-31762**

**McLaughlin et al, ‘Appropriate-for-gestational-age infants who exhibit reduced antenatal growth velocity display postnatal catch up growth’**

We thank the reviewer for their feedback and suggestions.

The line numbers, and much of the content, referenced by the reviewer belong to the original manuscript (submitted on 14^th^ November 2019), not to the subsequent revision, submitted on 12^th^ May 2020. Many of the points raised had thus already been addressed in this revision. We wonder whether it is possible that the reviewer received- or reviewed- the original submission in error rather than the revised version with all of our accompanying responses. Nevertheless, here we are pleased to again provide point-by-point responses to the feedback and submit this second revision of our manuscript for consideration.

For ease of review, we submit a manuscript with tracked changes, and also submit an identical clean copy. Where relevant, we have attempted to provide both the line number from the original manuscript, and the line numbers from (the tracked changes copy of) the previously submitted revision. We apologise that this is somewhat confusing, but it was the only way to address the comments of the reviewer which matched the line numbers of the original (not revised) version. We hope that the changes summarised here meet with the approval of the reviewers.

We look forward to your further correspondence,

Dr Emma McLaughlin

Corresponding author.

1. **This study reinforces the point that the common cut off for SGA births as <10th percentile is an arbitrary cut off that has limitations and observed that infants who were diagnosed with growth restriction during pregnancy grow at faster rates in their first 4 months of life**

To meet this request of the reviewer we previously added a line to the introduction in our first revision. This point is discussed where we state (page 4, lines 68-74):

*“UPI results in failing growth, commonly manifesting as low birthweight centile. As such, small-for-gestational-age (SGA; estimated fetal weight (EFW), or birthweight <10th centile) is the most commonly used proxy for FGR, as although not all SGA fetuses are growth restricted (some are constitutionally small), it does represent an at-risk cohort. Being SGA is the greatest risk factor for stillbirth (3) …”*

2. **The biggest concern with this manuscript is the acceptance of the Barker hypothesis, which is repeated numerous times throughout the manuscript. I encourage these authors to consider a critical appraisal of that hypothesis. Paragraph beginning on Line 80: The ‘Barker hypothesis’ (13) proposes that the relationship between FGR and adult disease is due to fetal programming in utero. There is good evidence to show that the ‘Barker hypothesis’ is not supported and due to over-adjustment in regression analysis (1-4), which is known to distort regression analysis results (5,6) so this section should be revised to mention that: the ‘Barker hypothesis’ is only a hypothesis, several mechanisms have been proposed for how slow followed by rapid growth might lead to adult medical issues, and how based on this hypothesis and evidence from studies that used the Barker regression methods suggest that catch-up growth could be part of the mechanism.

   1. Kramer MS, Zhang X, Dahhou M, Yang S, Martin RM, Oken E, et al. Does fetal growth restriction cause later obesity? Pitfalls in analysing causal mediators as confounders. Am J Epidemiol. 2017 Apr;185(7):585–90.
   2. Huxley R, Neil A, Collins R. Unravelling the fetal origins hypothesis: is there really an inverse association between birthweight and subsequent blood pressure? Lancet 2002 Aug;360(9334):659–65.
   3. Paneth N, Ahmed F, Stein AD. Early nutritional origins of hypertension: a hypothesis still lacking support. J Hypertens Suppl. 1996 Dec;14(5):S121–9.
   4. Tu Y-K, West R, Ellison GTH, Gilthorpe MS. Why evidence for the fetal origins of adult disease might be a statistical artifact: the “reversal paradox” for the relation between birth weight and blood pressure in later life. Am J Epidemiol. 2005;161:27–32.
   5. Schisterman EF, Cole SR, Platt RW. Overadjustment bias and unnecessary adjustment in epidemiologic studies. Epidemiology [Internet]. 2009;20(4):488–95.
   6. Ananth C V., Schisterman EF. Confounding, causality, and confusion: the role of intermediate variables in interpreting observational studies in obstetrics. Am J Obstet Gynecol [Internet]. 2017;217(2):167–75.’**

The reviewer raised this point in their review of the original manuscript, and we revised the manuscript accordingly. In light of these comments we wonder if the reviewer may have not received, or reviewed, the revised version of the manuscript.

While this study is based on belief in the fetal origins of adult health and disease theory (line 80 in the original manuscript, not in the revised version of the manuscript (this theory is now discussed from line 87 in the revised manuscript), this theory is not universally accepted. We believe we addressed this point in our first revision where we added a section to the strengths and limitations section of our discussion utilising the references suggested by the reviewer at time of their first review, which states (Page 19, lines 378-383):

*“Finally, this study interrogating fetal and postnatal growth rates is based upon the developmental origins of health and disease hypothesis. While this is a theory to which we ascribe, it is not universally accepted, with some research groups considering that the relationships between fetal growth and adult disease seen may be due to over-adjustment due to analysis method used (35-38)”*

We have not made any further changes, as we believe the reviewer may not have had the benefit of seeing the revisions made already.

3. **EFW growth velocity defined as a fall of 20 and 30 centiles in line 112 and 133. Please clarify.**

Again, the use of differing definitions of EFW growth velocity; loss of >30 centiles in the original FLAG study and loss of >20 centiles in this FLAG follow-up study (lines 112 and 133 in the original manuscript and line 119 and 149 in the revised manuscript, respectively) was addressed in our first revision. We used a broader cut-off of >20 centiles EFW loss instead of >30 centiles loss to ensure enough cases for reasonable statistical comparison, given that we expected loss to follow up. At the time of our first revision we added more detail regarding our use of the different cut-offs in the Methods section, page 7, lines 144-153 which reads:

***“****The current study focussed on comparing antenatal and postnatal growth trajectory, not predictive performance for adverse outcome for which the original study was powered. Given the different outcome being interrogated, and expected loss to follow up, we used a more sensitive cut-off for growth velocity than the original FLAG study to ensure a sufficient number of cases of low antenatal growth velocity. Fetuses who exhibited an antenatal decrease in EFW of >20 customised centiles over eight weeks were defined as experiencing low antenatal growth velocity and to potentially be growth restricted (labelled as AGA-FGR). The remainder of the AGA fetuses were defined as demonstrating normal antenatal growth (AGA-NG)."*

We have therefore not made any further changes, regarding this point.

4. **Catch-up growth is referred to negatively in lines 264, 282, 343, 376-7 and 393-4, which should be revised to remove the value judgements, especially given the large percentile anthropometric recoveries seen in this study (produced a distribution of sizes that were similar to the non-growth restricted infants) which suggests that the infants tend to grow in this manner. This negative messaging about catch-up growth would not be desirable to be presented to parents to have parents restrict infants’ feedings to prevent this catch up growth, as further suboptimal growth would not likely support good brain growth and it could harm the relationship between the parent(s) and the child. E.g. Line 264: the SGA and AGA-FGR cohorts were both found “to be at increased risk” of catch-up-growth when compared to the AGA-NG group - since catch up growth is not necessarily undesirable, I suggest this be changed to “have a higher probability”.**

We agree that catch-up-growth in not necessarily undesirable. As recognised in the introduction, it is thought to be beneficial for neurodevelopment (Page 4, lines 81-82). To meet the request of the reviewer regarding the language in lines 264, 282, 343, 376-7 and 393-4 in the original manuscript (lines 320, 338, 409-410, 446-447 and 461-463 in the revised manuscript, respectively) we have adjusted the wording as the reviewer suggested in these sentences so that they now read with a more neutral tone, as follows:

Page 16, lines 322-325:

*“When between pair testing was then performed, the SGA and AGA-FGR cohorts were both found to have a higher probability of catch-up growth when compared to the AGA-NG group (Table 3). For the SGA cohort the probability was approximately tripled, and for the AGA-FGR cohort, it was doubled.”*

Page 18, lines 343-344:

*“We have demonstrated that catch-up growth – hypothesised to be associated with some long-term health decrements”*

Page 21, lines 419-421:

*“While their findings support our results, we know that both SGA and preterm AGA infants are more likely to demonstrate catch-up growth (22, 43)”*

We elected not to change the language used in the final two sentences highlighted by the reviewer, as these speak directly to negative health outcomes, and therefore the word “risk” is appropriate in this context. We highlight however that these sentences both speak hypothetically using the word “may”. They read as follows (page and line numbers reference their location in this, the second revision):

Page 22, lines 461-463:

“*These infants may therefore be at increased risk of the antenatal, intrapartum, postnatal, childhood and adulthood complications that FGR and catch-up growth confer compared to AGA-NG infants”*

And conclusion, page 24, lines 485-487

“*Our findings suggest that these infants may also be at increased risk of developing the long-term health decrements traditionally associated with infants born SGA”*

5. **It would be desirable also to refer to the RR as the risk ratio.**

We have chosen to continue to refer to the RR as ‘relative risk’ for continuity of language with our previous studies.

6. **Lines 357-364 – while rapid weight gain has been associated with later obesity, these studies should be examined for whether or not they adjusted for the social determinants of health, since many social determinants can put people at risk of living in an obesogenic environment, and may be the cause of both early weight gain and later obesity.**

We acknowledge that there is an association between low socioeconomic status and low birth weight, as well as low socioeconomic status and obesity in developed countries. In lines 357-364 in the original manuscript (now corresponding to pages 21-22, lines 437-449), the referenced articles by Settler et al. (46) and Monteiro et al. (47) adjusted for socioeconomic status in their analyses. To make this point clear, lines 437-445 now read as:

*“Rotevatn et al. (45), Stettler et al. (46) and Monteiro et al. (47) found that rapid weight gain in the first year of life was associated with being overweight at 2, 7, and 14-16 years of age, respectively, independent of socioeconomic status. This is supported by a recent meta-analysis which found that the odds of being overweight from childhood to adulthood was 3.66 times greater in infants who experienced catch-up growth (48).”*

7. **This paper would be enhanced with the median measurements for all 3 groups for wt, L and HC plotted on the WHO growth charts. An App such as this could be used** [**https://apps.cpeg-gcep.net/growth02/**](https://apps.cpeg-gcep.net/growth02/)

The data analysis has largely been repeated after the first review of the original manuscript, and the reporting of our results along with it. Our figures now plot the predicted mean z-scores for weight, length and BMI for all three infant groups together – similar to the suggestion made by the reviewer here. We have elected not to plot graphs onto the WHO growth charts however as these plot centiles, and we now report z-scores only, and further, the app suggested for use does not allow us to plot the three groups together on one graph. Therefore, we have not altered the figure representation of our data from our first revision of the paper which were a major change at time of first review.

8. **The results could appear considerably differently if change in SD-scores were used instead of centiles, since SD-scores are linear across the normal curve distribution while centiles increment in a non-linear fashion. For example, between 0, 1, 2 and 3 SD-scores, there are 34%, 13%, 3% and <0.1% of the normal curve distribution**.

We already changed all of our reporting to SD scores (z-scores) at the time of the first revision. Given that only SD scores have been used in the manuscript since that time for assessment postnatal growth, we have not made any further changes to this point.

9. **“in the face of” = colloquial, not plain language**

The wording has been changed on page 4, lines 68-69 to read:

*“Decreased nutrient and oxygen supply consequent to UPI results in failing growth, commonly manifesting as a low birthweight centile”*

10. **178 recruitment bias: it would be preferable to use the words selection bias in the language used by the Cochrane Collaboration**

The wording has been changed on page 9, lines 189-192 which now reads:

*“Maternal characteristics and birth outcome data were compared between both: recruited follow-up study participants and the eligible women who did not participate, to check for selection bias; and cases of low antenatal velocity and the remainder of the AGA cohort, the subjects of our primary comparison”*

Again, in our results we have changed the wording (page 11, lines 232-233) to read:

*“We compared the maternal characteristics and delivery outcomes of the 156 women we recruited to the 189 non-responders to assess for selection bias (S1 Table).*

11. **Table S1: What are the units for EFW change in 8 weeks?**

The units for EFW change in 8 weeks has been added to Table S1

12. **Table S1: It is good practice to use 2 digits in the p-values on the right side of the** **decimal point for NS findings and 3 digits for significant findings.**

In light of this point, the number of decimal points presented for p-values in Table S1 are now consistent.

13. **Line 227: (-1.9 vs. -13.8, p=0.04): interesting – looks like the NG group had regression to the mean or some catch down growth**

Yes, we agree with the reviewer on this point which is why this finding is described in the manuscript on pages 14-15, lines 298-302:

*“…the shape of the growth curves in Figs 1 and 2 show that the AGA-FGR group demonstrated a similar, physiological fall in z-scores like the AGA-NG group, but of a lesser magnitude – leading to their catch-up growth. In contrast, the SGA cohort’s catch-up growth took the form of a steady increase in z-scores without the physiological fall seen among the AGA.”*

14. **Line 230 – missing a “p=”?**

The sentence previously containing the missing “p=" (previously line 230 in the original manuscript, not the first revision) no longer exists in the current (revised) manuscript.

15. **Line 232 – should be past tense: “occurs”,**

Line 232 (now page 13, line 272) was corrected to past tense in the previous manuscript revision and therefore has required alteration in this revision

16. **Line 351 – should be past tense**

Line 351 in the original manuscript (lines 417-419 in the first revision of the manuscript) now has now been corrected to the past tense on page 21, lines 428-430:

*“While catch-up growth was more evident in weight than length between birth and 4 months in our cohort, at no point in the first 2 years was BMI greater in AGA-FGR than in AGA-NG infants.”*

17. **How did the nurses assign the percentiles? Was it using growth charts or using a computer tool? Which was used will help to describe the precision or lack thereof**

Maternal child health nurses did not assign percentiles. Raw measurements of length, weight and head circumference were taken in centimetres to 1 decimal place or to the nearest gram for weight. We obtained the records of these measurements and converted them to z-scores using the WHO child growth standards software as described on pages 6 and 8 in lines 126-131 and 168-170 (transcribed below), respectively. This software is similar to that recommended by the reviewer in point number 5 (<https://www.who.int/childgrowth/software/en/>).

*“This collected… the infant weight, length and head circumference measurements. These measurements are routinely performed during maternal-child-health visits at 1, 2, 4 and 8 weeks; 4, 8, 12 and 18 months; and at 2 years of age and recorded in the child’s analogue ‘My Health, Learning & Development Record’…*

*Then “Using the WHO child growth standards software (29), we calculated standard deviation z-scores, adjusted for sex and age, for weight, length and body mass index (BMI; weight(kg)/height(m)^2^)”*
